# Polygenic scores for psychiatric disorders in a diverse postmortem brain tissue cohort

**DOI:** 10.1038/s41386-022-01524-w

**Published:** 2023-01-24

**Authors:** Laramie Duncan, Hanyang Shen, Anton Schulmann, Tayden Li, Bhaskar Kolachana, Ajeet Mandal, Ningping Feng, Pavan Auluck, Stefano Marenco

**Affiliations:** 1 Department of Psychiatry and Behavioral Sciences, Stanford University, Stanford, CA 94305 USA; 2 Wu Tsai Neuroscience Institute, Stanford University, Stanford, CA USA; 3 Epidemiology and Clinical Research Graduate Program, Stanford University, Stanford, CA USA; 4 Human Genetics Branch, NIMH-IRP, Bethesda, MD 20892 USA; 5 Human Brain Collection Core (HBCC), NIMH-IRP, Bethesda, MD 20892 USA

**Keywords:** Behavioural genetics, Genetic markers

## Abstract

A new era of human postmortem tissue research has emerged thanks to the development of ‘omics technologies that measure genes, proteins, and spatial parameters in unprecedented detail. Also newly possible is the ability to construct polygenic scores, individual-level metrics of genetic risk (also known as polygenic risk scores/PRS), based on genome-wide association studies, GWAS. Here, we report on clinical, educational, and brain gene expression correlates of polygenic scores in ancestrally diverse samples from the Human Brain Collection Core (HBCC). Genotypes from 1418 donors were subjected to quality control filters, imputed, and used to construct polygenic scores. Polygenic scores for schizophrenia predicted schizophrenia status in donors of European ancestry (*p* = 4.7 × 10^−8^, 17.2%) and in donors with African ancestry (*p* = 1.6 × 10^−5^, 10.4% of phenotypic variance explained). This pattern of higher variance explained among European ancestry samples was also observed for other psychiatric disorders (depression, bipolar disorder, substance use disorders, anxiety disorders) and for height, body mass index, and years of education. For a subset of 223 samples, gene expression from dorsolateral prefrontal cortex (DLPFC) was available through the CommonMind Consortium. In this subgroup, schizophrenia polygenic scores also predicted an aggregate gene expression score for schizophrenia (European ancestry: *p* = 0.0032, African ancestry: *p* = 0.15). Overall, polygenic scores performed as expected in ancestrally diverse samples, given historical biases toward use of European ancestry samples and variable predictive power of polygenic scores across phenotypes. The transcriptomic results reported here suggest that inherited schizophrenia genetic risk influences gene expression, even in adulthood. For future research, these and additional polygenic scores are being made available for analyses, and for selecting samples, using postmortem tissue from the Human Brain Collection Core.

## Introduction

Given the inaccessibility of the living human brain, biobanks containing brains from human donors are critical for psychiatric research. Interest in postmortem tissue samples has accelerated in recent years owing to improvements in ‘omics technologies which afford unprecedented detail in the measurement of not only genes and proteins, but also spatial parameters describing the density, patterning, and composition of neuronal structures. Thus, it is a promising time for data-driven discoveries about the neurobiology of psychiatric disorders.

Concurrent with the development of modern ‘omics technologies has been the discovery of many common genetic variants that influence risk for psychiatric disorders like schizophrenia [1, 2], depression [3], bipolar disorder [4], and PTSD [5], phenotypes which are now known to be highly polygenic. This genetic risk can be represented by calculating polygenic scores (also known as polygenic risk scores/PRS or genomic risk scores/GRS) which combine the effects of tens to hundreds of thousands of variants across the genome and are useful in quantifying individual risk for psychiatric disorders. Polygenic scores for schizophrenia are currently the most predictive among psychiatric disorders, explaining approximately 18% of phenotypic variance [2, 6]. Now it is possible to use these polygenic scores in analyses of postmortem tissue, and in the selection of samples for postmortem tissue projects, as we describe in this manuscript.

Polygenic scores are typically constructed using allelic weights derived from large-scale genome-wide association studies (GWAS), and consequently, they are available for a wide variety of quantitative traits such as height, body mass index (BMI), and years of education completed. The predictive performance of polygenic scores improves with increasing statistical power of the training GWAS because better powered GWAS afford more accurate estimates of the effect sizes of individual alleles, and therefore better polygenic prediction of disease status in independent samples [7, 8].

Polygenic scores are useful because they provide individual-level metrics of disease risk, but studies of the biological ramifications of polygenic scores are needed, and here we describe the construction of, and foundational analyses for, clinical and gene expression correlates of polygenic scores in a large human brain biobank. The Human Brain Collection Core (HBCC) is a brain bank within the National Institute of Mental Health (NIMH) Intramural Research Program. This collection is carefully curated with deep clinical, genotypic, toxicological, and neuropathological assessment [9]. The Human Brain Collection Core has previously contributed to large-scale genomics investigations including the CommonMind Consortium and PsychEncode, yielding novel findings about psychiatric diagnoses and genetic variants, RNA expression, and chromatin accessibility [9–14]. To further increase the utility of this resource, genotyping arrays were used to obtain genome-wide single nucleotide polymorphism (SNP) data for 1418 postmortem brains in the Human Brain Collection Core.

As an initial step toward leveraging postmortem data to identify the neurobiological correlates of polygenic risk for psychiatric disorders, we assessed the predictive performance of polygenic scores in Human Brain Collection Core samples and compared performance to the existing literature. We also prepared quantitative variables that are informative about relatedness and ancestry among HBCC samples for future studies. Finally, in a subset of subjects with gene expression data from the dorsolateral prefrontal cortex (DLPFC), we constructed an aggregate gene expression score (quantifying differential gene expression between schizophrenia cases and controls) and then tested the hypothesis that higher polygenic scores for schizophrenia were predictive of higher aggregate gene expression for schizophrenia in the HBCC.

## Patients and methods

### HBCC donors and postmortem brain tissue preservation

The HBCC collects brains primarily from medical examiners in Virginia and DC with the permission of the next of kin. Brains are sectioned in coronal slabs and flash frozen in a slurry of isopentane and dry ice and preserved at −80 C. Postmortem brains were from individuals with psychiatric disorders (including substance use) or individuals who did not have neuropsychiatric illness in their lifetime. A telephone interview with the next-of-kin was used to gather basic demographic information and medical history. Medical records were reviewed, and a consensus clinical diagnosis based on DSM-IV [15] or DSM5 [16] was reached by two psychiatrists. All cases were assessed by a neuropathologist and found free of degenerative disease. Procedures for brain collection and processing have been described in previous publications [9]. HBCC makes available their inventory of cases through the NIH NeuroBioBank (https://neurobiobank.nih.gov/). Some of the samples that constitute the HBCC collection of genotypes and gene expression data were provided by the University of Maryland Brain and Tissue Bank (https://www.medschool.umaryland.edu/btbank/) and the Stanley Medical Research Institute Brain Research Tissue Repository (https://www.stanleyresearch.org/brain-research/).

### Donor characteristics

**Fig. 1:**
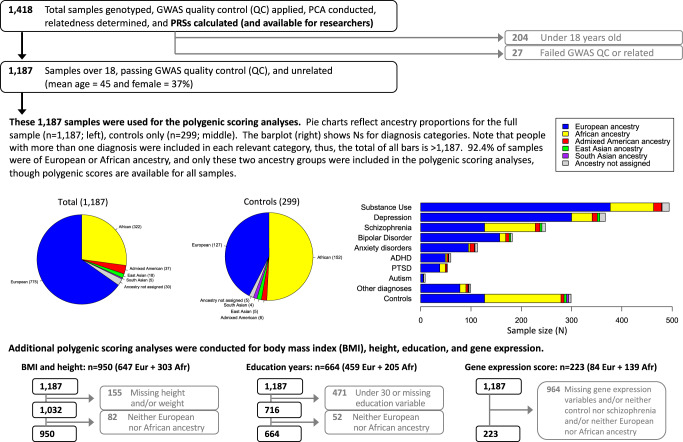
Flowchart of sample sizes by analysis step, ancestry, and diagnosis. Polygenic scores are available for all samples, but tests of polygenic scores were only applied to relevant sub-samples. Gray arrows and boxes denote samples excluded at various stages in the analysis pipeline. Eur European ancestry, Afr African ancestry.

1418 unique postmortem brains with genome-wide genotype data were available through the Human Brain Collection Core. As described below, we constructed polygenic scores for all 1418 samples. Of these individuals, 1187 were at least 18 years old, unrelated, and passed the genotyping quality control filters (37% female, mean age 45, filters described below). Demographics for these samples can be found in Fig. 1. 92% were of European or African ancestry and the remaining 8% of samples were divided among additional ancestry subsets. Due to sample size considerations, only European and African ancestry samples were used for polygenic scoring analyses (see below for further details). A further subset of 223 subjects with gene expression data from the dorsolateral prefrontal cortex were analyzed; see Fig. 1 for details.

Substance use disorders (including any substance abuse or dependence diagnosis) were the most common, followed by diagnoses of depression, schizophrenia, and bipolar disorder (Fig. 1). Polygenic scores for schizophrenia are the most predictive of case-control status in large GWAS studies, thus we focused on schizophrenia as our primary phenotype. However, we also provide polygenic scoring analyses for additional diagnostic categories with at least 100 individuals, namely: substance use disorders, depression, bipolar disorder, and anxiety disorders. See Table 1 for exact sample sizes by ancestry and diagnosis category. Psychiatric comorbidities are common in general and in these samples, and in the absence of any strong rationale for making exclusions based on comorbidities, we retained all individuals with a given diagnosis for the corresponding polygenic scoring analyses.

**Table 1:** Sample sizes for the 1187 samples that were over 18 and passed genotyping quality control.

	All	European	African	Admixed American	East Asian	South Asian	Not assigned
All samples: *n*	1187	775	322	37	18	5	30
Diagnosis categories: *n*							
Substance Use	494	377	86	16	1	0	14
Depression	367	300	41	10	5	0	11
Schizophrenia	248	127	101	9	4	0	7
Bipolar Disorder	182	157	12	7	3	0	3
Anxiety Disorders	113	95	3	9	1	0	5
ADHD	60	49	3	3	1	0	4
PTSD	53	38	11	3	0	0	1
Autism	9	5	1	0	0	0	3
Other Diagnoses	99	78	12	4	1	1	3
Controls	299	127	152	6	5	4	5
Quantitative phenotypes: *n* (mean, standard deviation)							
Height in inches	1032 (67.9 4.0)	647 (68.4 3.8)	303 (67.3, 3.9)	31 (66.0, 3.6)	17 (64.6, 4.9)	5 (63.3, 3.7)	29 (68.3, 4.7)
Body mass index	1032 (29.4, 8.3)	647 (28.5, 7.1)	303 (31.5, 10.1)	31 (29.8, 9.9)	17 (27.3, 5.3)	5 (26.1, 3.1)	29 (27.5, 7.2)
Education	716 (13.9, 3.0)	459 (14.4, 3.0)	205 (12.5, 2.6)	17 (13.9, 2.8)	12 (15.5, 3.2)	5 (16.2, 2.7)	18 (13.1, 3.0)
Gene expression							
Aggregate score	223	84	139	–	–	–	–

Sample sizes are given for all individuals (left-most column) and by ancestry. Note that ancestry subsets are mutually exclusive, but that diagnosis categories (rows) may not be mutually exclusive, but only in accordance with DSM-5 criteria. For example, DSM-5 does not allow simultaneous diagnosis of bipolar disorder and depression, and as such there are no individuals with these un-allowable co-morbidities. In contrast, simultaneous diagnosis of anxiety disorders and depression is allowable, and individuals with such comorbidities are included in each of the relevant cells in the table. The controls have no diagnoses, and therefore do not overlap with any diagnostic categories. For the quantitative phenotypes, mean and standard deviations are given in addition to sample sizes.

### Genome-wide genotyping via microarrays, quality control, relatedness, ancestry, and imputation

Genotyping was conducted in four batches using three different Illumina arrays (Human1M-Duov3_B, H650KHumanHap650Yv3.0, and HumanOmni5-Quad for two batches). Typical GWAS quality control procedures were applied to each of the four batches separately. Quality control, imputation, relatedness, and ancestry/principal components analysis steps are given in “HBCC_QCsteps_5_16_22_SHARED.xlsx”. SNPs and samples were excluded according to the following steps, applied sequentially: SNPs with missingness >5%, samples with variant missingness >2%, samples with deviation from expected inbreeding coefficient (*fhet* > |0.2 | ), SNPs with missingness >2%^1^, SNPs with Hardy Weinberg Equilibrium deviation *p* < 1 × 10^−6^, duplicated SNPs, and strand-ambiguous SNPs.

For each batch, genotypes were imputed to 1000Genomes phase3 v5 (genome build GRCh37/hg19) using the Michigan Imputation Server [17], with the following parameters: genotyping imputation = Minimac 4, input array build = GRCh37/hg19, rsq filter = off, phasing = Eagle v2.4 (phased output), population = other/mixed, mode = quality control & imputation. Imputed variants were filtered to retain biallelic SNPs and SNPs likely to have higher imputation accuracy (imputation r-squared >0.8) and to exclude strand ambiguous SNPs. These four datasets were then merged into a combined dataset with 1418 individuals and 8,874,206 SNPs; the PLINK format filenames with checksum values for verification are as follows:

HBCC_imputed_clean_4datasets_HY_01_15_2021.bed (e80b8c39e7acc153ac15702164e5a8d8)

HBCC_imputed_clean_4datasets_HY_01_15_2021.bim (de0cce7eb7ef490103a57ac4fad1260d)

HBCC_imputed_clean_4datasets_HY_01_15_2021.fam (8ad5febd0c1a98238a76cb397ad4eeff)

This merged dataset was used for the relatedness, ancestry, and polygenic scoring steps below.

### Relatedness and ancestry/principal components analysis

Relatedness and ancestry in this collection with diverse ancestral backgrounds were determined with relatedness- and admixture-aware software (PC-Relate [18] and PC-AiR [19]) along with the use of the 1000Genomes [20]. The workflow used three R [21] packages: GENESIS, SNPRelate, and GWASTools. For the following steps, PLINK [22, 23] files were converted to gds format.

In brief, variants were pruned using PCRelate with the following parameters: slide.max.bp = 10 × 10^−7^, ld.threshold = sqrt(0.1). Then a robust KING matrix was generated. Next, principal components were generated using PC-AiR. Then PC-Relate generated relatedness parameters for all pairs of individuals. Finally, we imposed the following recommended thresholds successively on the relatedness parameters to label putative relatives: monozygotic twins (kin^2^ > 0.45, k0 < 0.2), parent-offspring (kin > 0.24 and kin < 0.26, k0 < 0.01), full siblings (kin > 0.2 and kin < 0.3, k0 > 0.1 and k0 < 0.4), and second-degree relatives (kin > 2^−(9/2)^ and kin < 0.2, k0 > 0.23). Using these determinations, 20 samples had relatedness estimated to be second-degree relative or more with another subject, and these 20 individuals were removed from polygenic scoring analyses. Further investigation revealed that these twenty individuals included one pair of brothers, another pair of likely second-degree relatives, and 16 instances in which sample contamination was likely the cause of the apparent, but erroneous, relatedness.

**Fig. 2:**
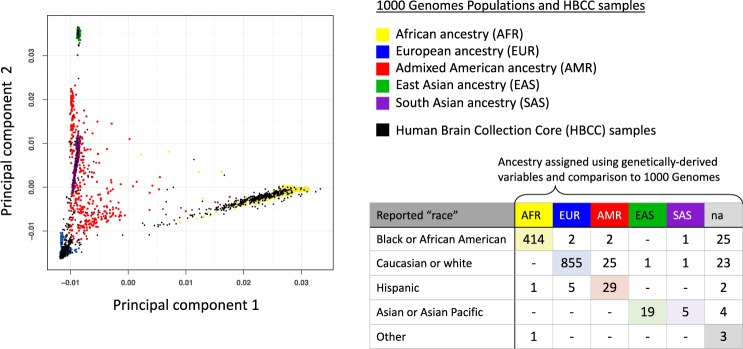
Ancestry-informative variables for Human Brain Collection Core (HBCC) donors. Principal components analysis was applied to a pruned set of SNPs from HBCC samples (*N* = 1418) and 1000 Genomes samples combined in order to infer major ancestry distinctions among of HBCC samples. As can be seen in the scatter plot, HBCC samples clustered with the major population groups from 1000 Genomes. Note that density of points is not clear from this plot and that the majority of the HBCC samples cluster with the 1000 Genomes European ancestry samples (blue) and African ancestry samples (yellow). The table (bottom right) shows that genetics-based ancestry assignments were broadly consistent with reported categories in the “race” variable from HBCC.

Assignment of broad ancestry categories was performed using procedures typical for genome-wide association studies (GWAS) by computing principal components on a combined dataset of the Human Brain Collection Core and 1000 Genomes [20] samples. As can be seen in Fig. 2, individuals clustered in principal component space with 1000 Genomes populations. The first and second principal components plotted in Fig. 1 afford visualization of the five major ancestry groups from the 1000 Genomes project [20], denoted with the five colors used by the 1000 Genomes project [20]. The majority of HBCC samples clustered with the European ancestry (blue) and African ancestry (yellow) samples. These two ancestry subsets were separately used in another round of principal components analysis to generate “ancestry-specific” principal components. Note that these ancestry variables are not meant to capture all aspects of ancestry and ethnicity that are relevant to a person’s identity or health, but rather they are necessary to appropriately adjust for ancestry in polygenic scoring analyses. Both the quantitative PCs (1–20) and the categorical ancestry variables are available for use with these brain tissue samples. Note also that these genetically-based ancestry categories overlap considerably with the “race” categories available from the Human Brain Collection Core (see Fig. 2, bottom right corner: *chi-sq* = 3040.1, *df* = 20, *p* < 2.2 × 10^−16^).

### Construction of polygenic scores

Polygenic scores were constructed using the classic and widely-used pruning and thresholding approach [1, 8]. To select SNPs for inclusion in the scores, we applied 13 *p*-value thresholds (*p*_*T*_) to the training GWAS ranging from genome wide significant SNPs only (*p*_*T*_ = *p* < 5 × 10^−8^) to all SNPs (*p*_*T*_ = *p* < / = 1.00). Consistent with prior use of *p*_*T*_ = 0.05 to benchmark the predictive performance of polygenic scores for schizophrenia [2, 6] and given widespread use of this alpha level, we specified *p*_*T*_ = 0.05, a priori, as the threshold for which we would report results in the text. Publicly available GWAS results from prior publications were used to construct polygenic scores for: schizophrenia [2], depression [3], neuroticism [24], bipolar disorder [4], ADHD [25], alcohol use [26], cannabis use [27], opioid use [28], anxiety (both case-control and factor score versions of analysis) [29], body mass index (BMI) [30], educational attainment [31], intelligence quotient [32], and height [33], and are available from the Human Brain Collection Core for approved requests.

### Gene expression/RNA-sequencing (RNAseq) data

Gene-level RNAseq count data (i.e., RSEM expected counts) from the dorsolateral prefrontal cortex (DLPFC) and metadata from the CommonMind Consortium were downloaded from synapse.org [14, 34]. The dataset consists of 374 unique postmortem cases, 223 of which met our inclusion criteria (first: all necessary variables for the gene expression analyses described below were available, second: samples were either control or schizophrenia diagnosis, and third: samples were either of European or African ancestry). The final sample sizes for the gene expression analyses were European ancestry (*n* = 84) and African ancestry (*n* = 139).

### Covariate adjustment and differential gene expression

Gene expression counts were processed using a weighted least-squares linear regression model via the limma-voom approach [35]. Covariates were selected based on significant correlation with the top 20 principal components of gene expression. For collinear variables associated with the same principal component, correlated variables were selected iteratively until no significant correlation was detected for the remaining variables. The final model used for covariate regression, for each gene, was:

$$	Gene\,expression\left( {logCPM} \right)\sim library\,batch + sex + age + effective\,mapping\,rate\\ 	+\, intergenic\,rate + RNA\,integrity\,number\left( {RIN} \right) + post - mortem\,interval\left( {PMI} \right) + pH$$

Covariate-adjusted residuals from the above model were then analyzed as follows to generate schizophrenia weights for each gene (i.e., the coefficients for schizophrenia below reflect log-fold changes in gene expression, for each gene):

$$Gene\,expression\,residuals\sim schizophrenia(case/control\,status)$$

### Aggregate gene expression scores for schizophrenia

To obtain an aggregate measure of transcriptional correlates of schizophrenia for each individual, we created a weighted sum of each individual’s covariate-adjusted gene expression values multiplied by the schizophrenia weights, using only the genes that had nominally significant differential gene expression in schizophrenia (as determined using the second model above).

Thus, the aggregate gene expression scores for schizophrenia, for each person, were calculated using this formula using all nominally significant genes, *g*:

$${{{{{{{\mathrm{Aggregate}}}}}}}}\,{{{{{{{\mathrm{gene}}}}}}}}\,{{{{{{{\mathrm{expression}}}}}}}}\,{{{{{{{\mathrm{score}}}}}}}} =	 \mathop {\sum}\limits_{i = 1}^g gene\,expression\,residual_i \\ 	\ast schizophrenia\,coefficient_i$$

Therefore, a positive aggregate gene expression score indicates differentially expressed genes deviating from the mean in the direction of schizophrenia cases while a negative score indicates changes in the direction of controls. The aggregate gene expression score is intended as a global measure of deviance from normal for each case with schizophrenia, it is calculated in a similar fashion to the polygenic score and is therefore an appropriate transcriptomic correlate of global genetic risk.

### Polygenic prediction of psychiatric phenotypes, traits, and gene expression scores

Regression was used to test for relationships between polygenic scores and phenotypes (logistic for binary phenotypes and linear for quantitative phenotypes). Ten genotype principal components (per ancestry subset) were used to adjust for ancestry.

### Data availability and critical recommendations for future use

One of the goals of this work was to make polygenic scores for the Human Brain Collection Core brains available to researchers, so that sample requests can be based on genetic risk. To this end, researchers may request from HBCC the polygenic and gene expression score file (“1418_HBCC_genetic_variables_10_26_22.xlsx”), which contains polygenic scores for 14 phenotypes, with weights from the best-powered GWAS of each phenotype available at this time (schizophrenia [2], depression [3], bipolar disorder [4], anxiety disorders with two versions of outcome phenotype [29], alcohol use disorder [26], cannabis use disorder [27], opioid use disorder [28], ADHD [25], neuroticism [24], body mass index = BMI [30], educational attainment [31], intelligence quotient [32], and height [33]). For each of these phenotypes, polygenic scores based on 13 *p*-value thresholds are included. Also provided are two sets of genotype principal components: PCs for samples with ancestry similar to the 1000Genomes European ancestry populations (“PC_EUR” variables, *N* = 862) and PCs for samples with ancestry similar to the 1000Genomes African ancestry populations (“PC_AFR” variables, *N* = 416). Original genotypes are available from dbGaP and NDA as follows: dbGaP Study Accession: phs000979.v3.p2 (https://www.ncbi.nlm.nih.gov/projects/gap/cgi-bin/study.cgi?study_id=phs000979.v3.p2); NIMH Data Archive (NDA) collection#: 3151 (https://nda.nih.gov/edit_collection.html?id=3151). The polygenic risk scores, ancestry designations, and details of quality control steps will also be available in the same NDA collection.

The most critical consideration for use of the polygenic scores is ancestry given that polygenic scores are oftentimes highly correlated with ancestry, and therefore spurious findings may be generated if ancestry is not adjusted for correctly in analyses [36]. Presently, no methods are available that allow one to include individuals with diverse global ancestries in polygenic scoring analyses together, though this is desirable and is an important area of ongoing research.

## Results

### Polygenic predictions of donor psychiatric phenotypes

**Fig. 3:**
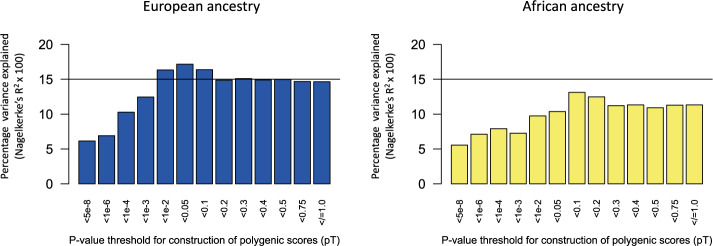
Schizophrenia polygenic score predictions of schizophrenia case status in the Human Brain Collection Core (HBCC). Bars in each plot reflect 13 p-value thresholds (*p*_*T*_) used to construct polygenic scores ranging from *p*_*T*_ = *p* < 5 × 10^−8^ (left most bars; only genome-wide significant variants) to *p*_*T*_ = *p* < /=1 (right most bars; all variants). Predictive performance was higher in the samples of European ancestry (left) as compared to samples with African ancestry (right), consistent with the lack of well-powered schizophrenia GWAS in African ancestry samples and differences in allele frequencies and linkage disequilibrium patterns across population gradients. Horizontal lines denoting 15% of phenotypic variance explained are provided for reference. All predictions survived multiple testing correction for 13 *p*_*T*_ values: p < .0038.

Schizophrenia polygenic scores were statistically significant predictors of schizophrenia case status in Human Brain Collection Core samples of European ancestry (*n* = 254) and samples with African ancestry^3^ (*n* = 253). Using the a priori selected *p*-value threshold (*p*_*T*_ = 0.05) and the largest schizophrenia GWAS [2] to construct scores, 17.2% of phenotypic variance was explained in the samples of European ancestry (*p* = 4.7 × 10^−8^), and 10.4% of phenotypic variance was explained in the samples with African ancestry (*p* = 1.6 × 10^−5^), and see Fig. 3.

Of note, *p*_*T*_ = 0.1, and not our a priori specified *p*-value threshold (*p*_*T*_ = 0.05), afforded the highest predictive performance in the samples with African ancestry (13.1% of phenotypic variance explained). Numerical results for all 13 *p*-value thresholds, for both ancestry subsets, are provided in Supplementary Table 1. As a negative control, we also attempted to predict schizophrenia with height polygenic scores; polygenic scores for height were not even nominally significant predictors of schizophrenia case/control status (e.g., *p* = 0.67, European ancestry subsample, *p*_*T*_ = 0.05). We also tested how well polygenic scores for bipolar disorder and depression predicted schizophrenia case control status in these samples; as expected, considerably less phenotypic variance was explained (3.6% and 1.7%, respectively, for European ancestry samples). Supplementary Fig. 1 shows polygenic prediction results within and across disorders for schizophrenia, bipolar disorder, and depression.

**Fig. 4:**
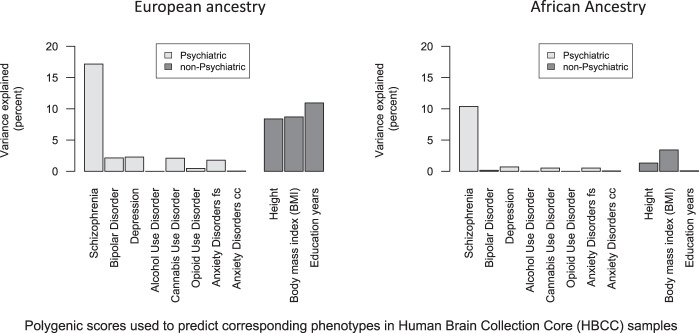
Phenotypic variance explained by corresponding polygenic scores in the Human Brain Collection Core (HBCC). Results for samples of European ancestry are on the left, and samples with African ancestry are on the right. Light gray denotes psychiatric phenotypes and dark gray denotes non-psychiatric phenotypes. Schizophrenia polygenic scores are the most powerful (left-most bars in both plots). Polygenic scores explain more phenotypic variance in European ancestry samples than samples with African ancestry. Note that three substance use polygenic scores (for alcohol, cannabis, and opioid phenotypes) were used to predict the same aggregate substance use phenotype in the HBCC samples. Two polygenic scores for anxiety were used (fs = factor score, cc = case control).

Polygenic prediction results for depression explained less variance and p-values were less significant, as compared to those for schizophrenia (see Supplementary Table 2 for full results predicting depression in European ancestry samples (*n* = 427, left) and samples with African ancestry (*n* = 193, right) for all *p*-value thresholds (*p*_*T*_) used to construct depression polygenic scores using the most powerful depression GWAS available at the time [3]). Using our a priori p-value threshold (*p*_*T*_ = 0.05), depression polygenic scores were nominally significant predictors of depression in European ancestry samples (*p* = 0.026, variance explained = 1.7%). Post-hoc examination of polygenic prediction results in the European ancestry samples revealed the highest variance explained was with *p*_*T*_ = 0.2 (*p* = 0.002, variance explained = 3.1%). For the samples with African ancestry, none of the polygenic prediction results were significant, but note that there were only 41 cases with depression in this analysis. See Fig. 4 for comparison of polygenic score predictive performance across phenotypes in Human Brian Collection Core samples. Note that the height of bars corresponds to phenotypic variance explained for polygenic scores constructed with *p*_*T*_ = 0.05, for each phenotype.

Full polygenic scoring results are available in Supplementary Tables 3–11 for prediction of bipolar disorder, substance use disorders, anxiety disorders, height, body mass index (BMI), and years of education completed. Regarding bipolar disorder, bipolar disorder polygenic scores were significant predictors of bipolar disorder status in the European ancestry subset (*p* = 0.03, variance explained = 2.1%, for *p*_*T*_ = 0.05 with weights from Stahl et al. 2019). Within the African ancestry subsample, only 12 subjects were identified as having bipolar disorder, and therefore it is not surprising that polygenic prediction was not significant (*p* = 0.73 for *p*_*T*_ = 0.05 with weights from Stahl et al 2019), see Supplementary Table 3 for full results. Similarly, many of the analyses reported in the Supplementary Tables are underpowered and consequently many of the results are not statistically significant. Despite low power in many of these analyses, the point estimates of variance explained by each polygenic score are nevertheless unbiased, and therefore we show comparative performance in Fig. 4 for these different polygenic scores.

Regarding polygenic prediction of quantitative phenotypes in the HBCC samples, we observed statistically significant prediction of height, body mass index (BMI), and years of education in the European ancestry subsample (8.4%, 8.7%, 10.9% of phenotypic variance explained, respectively; all *p* < 1.1 x 10^−13^, using *p*_*T*_ = 0.05). Among samples with African ancestry, polygenic predictions of height and BMI were nominally significant (*r*^* 2*^ = 1.3, *p* = 0.04, *n* = 303 and *r*^* 2*^ = 3.4, *p* = 0.001, *n* = 303, respectively), but polygenic prediction of years of education was not (*p* = 0.67, *n* = 205). For full results see Supplementary Tables 9–11.

### Polygenic prediction of an aggregate gene expression score for schizophrenia

**Fig. 5:**
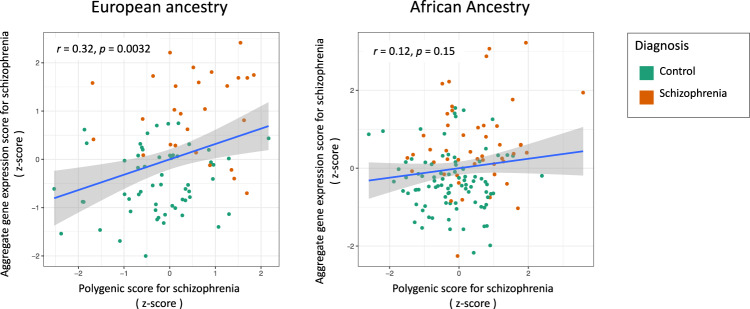
Positive correlation between polygenic score for schizophrenia and aggregate gene expression score for schizophrenia. Samples of European ancestry are shown on the left (*n* = 85) and samples with African ancestry are on the right (*n* = 139). Points represent individual donors color coded by diagnosis. The schizophrenia polygenic scores were constructed using weights from independent samples, and the aggregate gene expression scores were constructed using weights from the Human Brain Collection Core samples (see methods), with gene expression values from dorsolateral prefrontal cortex (DLPFC).

Regarding gene expression correlates of schizophrenia polygenic scores, we analyzed data from the subset of samples that had gene expression data available from the dorsolateral prefrontal cortex (DLPFC), including the samples that had a diagnosis of schizophrenia or were a control. Polygenic scores were correlated with aggregate gene expression scores in samples of European ancestry (*n* = 84, *r* = 0.30, *p* = 0.0055). Though not statistically significant, the point estimate of the correlation was also positive in the samples with African ancestry (*n* = 139, *r* = 0.12, *p* = 0.15) (Fig. 5). In Fig. 5, results are presented for correlations between ancestry adjusted polygenic scores and covariate adjusted gene expression scores to facilitate graphical representation. See Supplementary Table 12 for results across a range of principal components included to adjust for ancestry and for results across the 13 *p*-value thresholds (*p*_*T*_) for polygenic score construction.

## Discussion

Here, we demonstrate that polygenic scores for schizophrenia, depression, bipolar disorder, height, BMI, and years of education predict those phenotypes in Human Brain Collection Core samples with comparable performance to benchmark data sets.

The phenotype best accounted for by polygenic scores was schizophrenia (up to 17% of phenotypic variance explained), followed by the quantitative phenotypes of years of education, BMI, and height. For other psychiatric disorders, polygenic scores explained a smaller proportion of the variance (e.g., in European ancestry individuals, the best performing scores explained just 3.1% of variance in depression case/control status, comparable to the largest published investigation which reported 1.5% to 3.2% of variance explained in European ancestry datasets [3]).

A variety of factors likely explain the better performance of the polygenic scores for schizophrenia, height, BMI, and years of education as compared to polygenic scores for other psychiatric disorders. First, the discovery GWAS for these better-performing polygenic scores are more powerful than available GWAS for other psychiatric disorders. Two good indicators of power in discovery GWAS are the significance of the SNP heritability estimate (i.e., *h*^*2*^_SNP_ estimates from GCTA [37] and/or LDSC [38]) and the number of significant loci. As power for GWAS^4^ of a given phenotype increases, the SNP heritability estimate tends to become more significant, and the number of significant loci tends to increase. Thus, consumers of the literature may use a significant SNP heritability estimate combined with ten or more significant loci as a crude indicator of a GWAS being minimally adequately powered. Note that most of the polygenic scores used here that had low predictive power had few or no significant loci in the discovery GWAS (e.g., anxiety [29], opioid use [28]). A second consideration for the predictive performance of polygenic scores is the reliability or “robustness” of the phenotype. For example, schizophrenia is the most severe, disabling, and enduring amongst the psychiatric disorders analyzed here, while major depression is more episodic in course and can be comorbid and share more criteria with other psychiatric disorders. As a case in point, the GWAS of depression [3] used here achieved such a large sample size by including phenotypes ranging from subjective reports of depression to clinically ascertained major depression. This illustrates how variable psychiatric phenotype assessment can be and speaks to the heterogeneity of certain psychiatric diagnoses. Finally, given the relatively small sample sizes for certain phenotypic subsets used in the polygenic scoring analyses reported here, we expected that the percent of phenotypic variance explained might be highly variable for psychiatric phenotypes including bipolar disorder and anxiety disorders.

Beyond the well-documented reasons why polygenic scores perform worse in non-European ancestry samples [39–42], the results presented here are consistent with low power due to the small number of cases of African ancestry with depression (*N* = 41) and bipolar disorder (*N* = 12) in this collection. Further, it is possible that diagnoses were less reliable in the samples with African ancestry. Consistent with previously described diagnostic bias toward diagnosing Black individuals as having schizophrenia [43] and white individuals with similar symptoms as having the more “hopeful” diagnosis of bipolar disorder, in the Human Brain Collection Core, bipolar disorder accounted for just 10.6% of combined schizophrenia and bipolar disorder cases among samples with African ancestry, but 55.2% of combined schizophrenia and bipolar disorder cases among samples of European ancestry (*X*^2^ = 64.1, *df* = 1, *n* = 397, *p* = 1.2 x 10^−15^). This much higher proportion of schizophrenia cases among African ancestry samples could be partially due to diagnostic bias. Note, however, that diagnostic bias was not found to be the likely explanation in a different study that examined this issue [44].

These polygenic scores may be used in future studies of postmortem tissue from the Human Brain Collection Core. Polygenic scores for other phenotypes have also been constructed (for a total of 14 phenotypes: schizophrenia, depression, bipolar disorder, anxiety disorders with two versions of outcome phenotype, alcohol use disorder, cannabis use disorder, opioid use disorder, ADHD, neuroticism, body mass index, educational attainment, intelligence quotient, and height) and may be used for sample selection when requesting tissue from the Human Brain Collection Core. Ancestry-aware methods are critical for analyses using polygenic scores, and ancestry-specific genotype principal components have been provided for this purpose.

Beyond simple demonstration of the predictive performance of polygenic scores, we also sought to understand biological ramifications of higher and lower polygenic scores for schizophrenia. We found that polygenic scores for schizophrenia were positively correlated with aggregate gene expression schizophrenia scores. While correlational in nature, these findings suggest that inherited polygenic risk for schizophrenia influences brain gene expression and possibly other molecular phenotypes, even in adulthood.

Our results are consistent with weak but significant relationships described in the literature between genetic risk and transcriptomic features of schizophrenia [45]. The magnitude of the correlation observed here is similar to that found by Radulescu et al. [46] between schizophrenia polygenic scores and module eigengene expression, a more narrowly defined measure based on gene co-expression pattern. The magnitude of the correlation observed here between polygenic scores for schizophrenia and aggregate gene expression scores for schizophrenia was greater than that a previously reported correlation between polygenic scores and a co-expression module enriched in enhancer RNAs differentially expressed between patients with schizophrenia and controls [47]. The fact that several studies, including ours, find a relationship between polygenic scores and gene expression illustrates the utility of polygenic scores in postmortem studies of psychiatric populations. Polygenic scores are constructed completely independently of transcriptomic data and further were based on entirely independent samples from the ones studied here. Being able to link genotype and phenotype is critical to our interpretation of phenotypic differences between groups. While polygenic scores calculated here are a global measure of genetic risk, and therefore have poor biological specificity (a limitation of this study), polygenic scores can be computed on a more limited set of genes considered of biological relevance (e.g., genes belonging to a specific pathway or expressed in a specific cell type). More research is needed to assess whether such a strategy would be more biologically informative. The same applies to molecular phenotypes such as the aggregate gene expression score. Others have used more tailored transcriptional severity scores to gain increased biological specificity (e.g., in studies of schizophrenia [48], pneumonia [49], and systemic sclerosis [50]).

Prior work has established that most individuals with high polygenic risk scores for schizophrenia never develop schizophrenia, and this was also observed in these Human Brain Collection Core samples. For example, a population-based sample in Denmark was used to test for differences in likelihood of developing schizophrenia as a function of deciles of polygenic scores for schizophrenia. Those in the highest decile had approximately 8-fold higher risk of developing schizophrenia as compared to those in the lowest decile [51]. Indeed, it is interesting to note that the control individual with the highest schizophrenia polygenic risk had a nearly average aggregate gene expression score for schizophrenia, suggesting that protective factors could have helped to make this individual’s gene expression profile more typical (and perhaps more resilient to developing schizophrenia). Further work leveraging these polygenic scores and associated brain tissue samples may help to reveal why some individuals are resilient to the development of schizophrenia despite having relatively high polygenic risk. As well, much of the genetic risk for schizophrenia (with estimated 80% heritability) is not captured by these polygenic scores, and better quantification of genetic risk for schizophrenia and other psychiatric disorders awaits better discovery GWAS and sequencing studies, plus improved statistical methods. Indeed, we plan to make updated scores available periodically in the future, using GWAS with greater ancestral diversity as well as methods recently published [52] which maximize prediction across different ancestries.

These results pertained to gene expression in the dorsolateral prefrontal cortex, and future studies should examine further nuances of these findings. For example, it would be useful to understand cell type and regional differences in the putative effects of polygenic risk on gene expression. Developmental changes in gene expression are also dramatic, and future work can examine how polygenic risk for schizophrenia correlates with gene expression during brain development. Eventually, polygenic scores based on a restricted gene set (e.g., one pathway) that is biologically informative may be isolated and examined in brain gene expression. Finally, gene expression is just one potential process influenced by polygenic risk for schizophrenia, and future work can determine how cellular structures (e.g., dendritic spines, synapses, receptors), their patterning, and their functioning within specific brain circuits, may vary with inherited genetic risk.

## Supplementary information

Supplementary Figure 1

Supplementary Tables 1–12

## Footnotes

1 The second more stringent SNP missingness filter is intentional, after the initial less stringent SNP missingness filter and then removal of samples with missingness >2%.

2 kin = estimated kinship coefficient (0 = unrelated, 0.5 = monozygotic twins, 0.25 = parent offspring and full siblings); k0 = IBD coefficient, meaning the probability of sharing zero alleles identical by descent.

3 Many of the “samples with African ancestry” also had some European ancestry, as can be seen in Fig. 1.

4 Note that “GWAS” are actually usually meta-analyses of component GWASs (in order to achieve greater statistical power), but are often referred to as simply “GWAS”.
