# Supplementary material for: Polygenic scores for psychiatric disorders in a diverse postmortem brain tissue cohort
Source: Neuropsychopharmacology. 2023 Jan 24;48(5):764–72. doi: 10.1038/s41386-022-01524-w (PMC10066241; doi:10.1038/s41386-022-01524-w)

Supplementary Figure 1. Polygenic prediction results within and across disorders for schizophrenia, bipolar disorder, and depression. SCZ=schizophrenia, BIP=bipolar disorder, MDD=depression

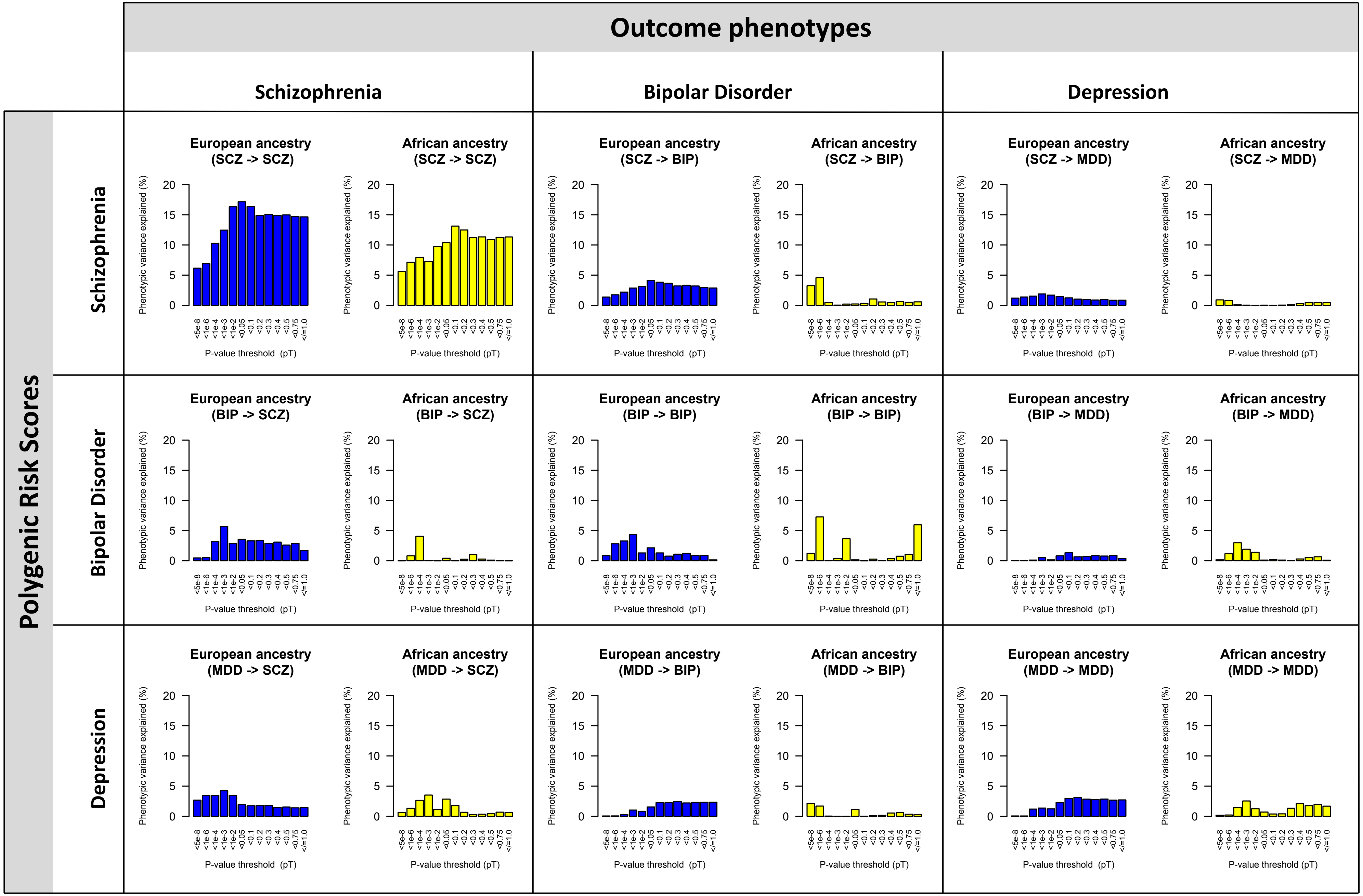

Supplement: Supplementary file 1 — Supplementary Figure 1 [file 41386_2022_1524_MOESM1_ESM.pdf]
